# 2,2,4-Trimethyl-5-(4-tolyl­sulfon­yl)-2,3,4,5-tetra­hydro-1*H*-1,5-benzo­diazepine

**DOI:** 10.1107/S1600536809034837

**Published:** 2009-09-05

**Authors:** K. Ravichandran, K. Sathiyaraj, S. S. Ilango, S. Ponnuswamy, M. N. Ponnuswamy

**Affiliations:** a Centre of Advanced Study in Crystallography and Biophysics, University of Madras, Guindy Campus, Chennai 600 025, India; b Department of Chemistry, Government Arts College (Autonomous), Coimbatore 641 018, India

## Abstract

In the title compound, C_19_H_24_N_2_O_2_S, the benzodiazepine ring adopts a distorted boat conformation. The S atom shows a distorted tetra­hedral geometry, with the O—S—O [119.16 (14)°] and N—S—C [107.48 (10)°] angles deviating significantly from ideal values. The crystal packing is controlled by C—H⋯O, N—H⋯O and C—H⋯π inter­actions.

## Related literature

For the use of benzodiazepines in the treatment of gastrointestinal and central nervous system disorders, see: Rahbaek *et al.* (1999 ▶). For hydrogen-bond motifs, see: Bernstein *et al.* (1995 ▶). For puckering and asymmetry parameters, see: Cremer & Pople (1975 ▶);) ; Nardelli (1983 ▶). For the Thorpe-Ingold effect, see: Bassindale (1984 ▶). For details of the preparation, see: Ponnuswamy *et al.* (2006 ▶).
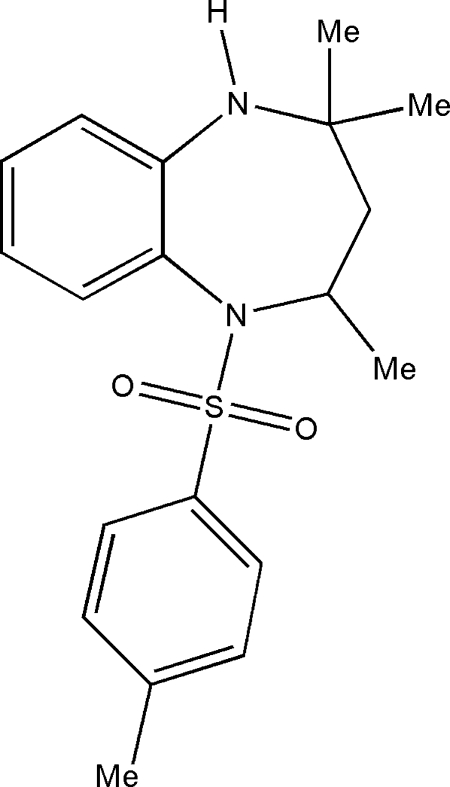


## Experimental

### 

#### Crystal data

C_19_H_24_N_2_O_2_S
*M* *_r_* = 344.46
Orthorhombic, 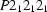

*a* = 7.3658 (3) Å
*b* = 14.8013 (8) Å
*c* = 17.4556 (10) Å
*V* = 1903.07 (17) Å^3^
*Z* = 4
Mo *K*α radiation
μ = 0.18 mm^−1^
*T* = 293 K
0.25 × 0.20 × 0.20 mm

#### Data collection

Bruker Kappa APEXII area-detector diffractometer
Absorption correction: multi-scan (*SADABS*; Sheldrick, 2001 ▶) *T*_min_ = 0.957, *T*_max_ = 0.964
13544 measured reflections
5154 independent reflections
3278 reflections with *I* > 2σ(*I*)
*R*_int_ = 0.034

#### Refinement

*R*[*F* ^2^ > 2σ(*F* ^2^)] = 0.047
*wR*(*F* ^2^) = 0.145
*S* = 1.04
5154 reflections
224 parameters
H atoms treated by a mixture of independent and constrained refinement
Δρ_max_ = 0.18 e Å^−3^
Δρ_min_ = −0.19 e Å^−3^
Absolute structure: Flack (1983 ▶), 2171 Friedel pairs
Flack parameter: −0.12 (9)

### 

Data collection: *APEX2* (Bruker, 2004 ▶); cell refinement: *SAINT* (Bruker, 2004 ▶); data reduction: *SAINT*; program(s) used to solve structure: *SHELXS97* (Sheldrick, 2008 ▶); program(s) used to refine structure: *SHELXL97* (Sheldrick, 2008 ▶); molecular graphics: *ORTEP-3* (Farrugia, 1997 ▶); software used to prepare material for publication: *SHELXL97* and *PLATON* (Spek, 2009 ▶).

## Supplementary Material

Crystal structure: contains datablocks global, I. DOI: 10.1107/S1600536809034837/bt5042sup1.cif

Structure factors: contains datablocks I. DOI: 10.1107/S1600536809034837/bt5042Isup2.hkl

Additional supplementary materials:  crystallographic information; 3D view; checkCIF report

## Figures and Tables

**Table 1:** Hydrogen-bond geometry (Å, °)

*D*—H⋯*A*	*D*—H	H⋯*A*	*D*⋯*A*	*D*—H⋯*A*
C20—H20⋯O2	0.93	2.51	2.885 (4)	105
N1—H1⋯O2^i^	0.75 (3)	2.52 (3)	3.268 (3)	176 (3)
C14—H14*B*⋯O1^ii^	0.96	2.58	3.436 (4)	149
C13—H13*A*⋯*Cg*2	0.96	2.90	3.7592 (31)	150
C19—H19⋯*Cg*2^i^	0.93	2.90	3.5192 (34)	126

Symmetry codes: (i) 
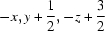
; (ii) 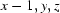. *Cg*2 is the centroid of the = C15–C20 ring.

**Fig. 1.**
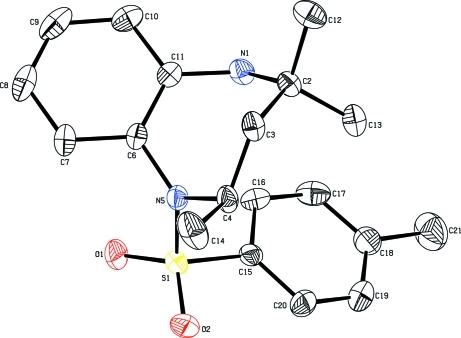
Perspective view of the molecule showing the displacement ellipsoids at 30% probability level. H atoms have been omitted for clarity.

**Fig. 2.**
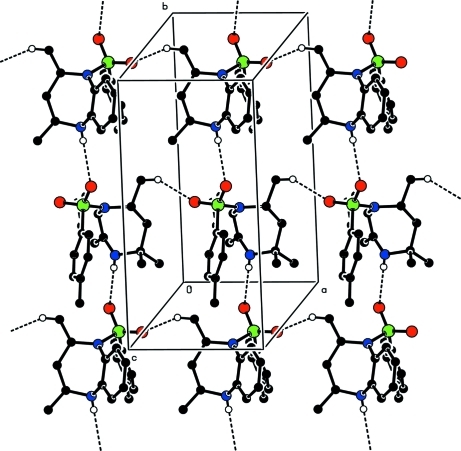
The crystal packing of the molecules viewed down c–axis. H atoms not involved in hydrogen bonding have been omitted for clarity.

## supplementary crystallographic information

### Comment

Benzodiazepines are known for their natural occurrence in filamentous fungi and actinomycetes of the *genera pencillium, aspergillus and streptomyces*. Benzodiazepines from *aspergillus* include *asperlicin*, which is used for treatment of gastrointestinal and central nervous system (*CNS*) disorders (Rahbaek *et al.*, 1999). In view of these importance and to ascertain the molecular conformation, a crystallographic study of the title compound has been carried out.

The *ORTEP* diagram of the title compound is shown in Fig.1. The benzodiazepine ring adopts a distorted boat conformation. The puckering parameters (Cremer & Pople, 1975) and the asymmetry parameters (Nardelli, 1983) for this ring are q_2_ = 0.976 (3) Å, q_3_ = 0.068 (3) Å, φ_2_ = 152.5 (2)°, φ_3_ = 30.0 (1)° and Δ2(C4)=7.8 (1)°. Atom S takes up a distorted tetrahedral geometry, with the O—S—O and N—S—C angles deviating significantly from ideal values, and this may be attributed to the Thorpe-Ingold effect (Bassindale, 1984). The sum of the bond angles at N1 (342.9°) of the benzodiazepine ring is in accordance with *sp*^3^ hybridization, whereas the one at N5(359.7°) is in *sp*^2^ hybridization, respectively.

The crystal packing is controlled by C—H···O, N—H···O and C—H···π types of intra and intermolecular interactions. Atom N1 at (*x*, *y*, *z*) donates a proton to O1 (-*x*, *y* + 1/2, -*z* + 1/2 + 1), which forms a one-dimesional C7 chain (Bernstein *et al.*, 1995) running along b–axis. The intermolecular hydrogen bond C14—H14B···O1 connects the molecule into a C6 one dimensional chain running along a–axis as shown in Fig 2. The methyl group H atom in the benzodiazepine ring interacts with the centroid atom of the toluenesulfonyl ring through an intramolecular C—H···π interaction involving atom C13, the separation between H13A and the centroid (*Cg*2) of the ring (C15/C16/C17/C18/C19/C20) being 2.899 Å.

### Experimental

To a solution of 2,2,4-Trimethyl-1*H*-tetrahydro-1,5-benzodiazepine (0.59 g) in anhydrous benzene (16 ml), triethylamine (0.7 ml) and 4-toluenesulfonyl chloride (1 gm) were added. The reaction mixture was allowed to reflux for 16 hrs. The benzene solution was washed with water, dried over anhydrous Na_2_SO_4_ and concentrated. The resulting mass was purified by crystallization from benzene (Ponnuswamy *et al.*, 2006).

### Refinement

The amino H atom was freely refined. The other H atoms were positioned geometrically (C—H=0.93–0.98 Å) and allowed to ride on their parent atoms, with 1.5*U*_eq_(C_methyl_)  or 1.2 *U*_eq_(C). *PLATON* (Spek, 2009) detected a solvent accessible void of approximately 31 Å^3^. This void could have initially contained solvent molecules but these molecules have since evaporated from the structure without degradation of the crystal.

### Crystal data

**Table:**

C_19_H_24_N_2_O_2_S	*F*(000) = 736
*M**_r_* = 344.46	*D*_x_ = 1.202 Mg m^−^^3^
Orthorhombic, *P*2_1_2_1_2_1_	Mo *K*α radiation, λ = 0.71073 Å
Hall symbol: P 2ac 2ab	Cell parameters from 2546 reflections
*a* = 7.3658 (3) Å	θ = 1.8–29.4°
*b* = 14.8013 (8) Å	µ = 0.18 mm^−^^1^
*c* = 17.4556 (10) Å	*T* = 293 K
*V* = 1903.07 (17) Å^3^	Block, colourless
*Z* = 4	0.25 × 0.20 × 0.20 mm

### Data collection

**Table:**

Bruker Kappa APEXII area-detector diffractometer	5154 independent reflections
Radiation source: fine-focus sealed tube	3278 reflections with *I* > 2σ(*I*)
graphite	*R*_int_ = 0.034
ω and φ scans	θ_max_ = 29.4°, θ_min_ = 1.8°
Absorption correction: multi-scan (*SADABS*; Sheldrick, 2001)	*h* = −5→10
*T*_min_ = 0.957, *T*_max_ = 0.964	*k* = −20→18
13544 measured reflections	*l* = −23→23

### Refinement

**Table:**

Refinement on *F*^2^	Secondary atom site location: difference Fourier map
Least-squares matrix: full	Hydrogen site location: inferred from neighbouring sites
*R*[*F*^2^ > 2σ(*F*^2^)] = 0.047	H atoms treated by a mixture of independent and constrained refinement
*wR*(*F*^2^) = 0.145	*w* = 1/[σ^2^(*F*_o_^2^) + (0.0742*P*)^2^] where *P* = (*F*_o_^2^ + 2*F*_c_^2^)/3
*S* = 1.04	(Δ/σ)_max_ = 0.009
5154 reflections	Δρ_max_ = 0.18 e Å^−^^3^
224 parameters	Δρ_min_ = −0.19 e Å^−^^3^
0 restraints	Absolute structure: Flack (1983), 2171 Friedel pairs
Primary atom site location: structure-invariant direct methods	Flack parameter: −0.12 (9)

### Special details

**Table:**

Geometry. All e.s.d.'s (except the e.s.d. in the dihedral angle between two l.s. planes) are estimated using the full covariance matrix. The cell e.s.d.'s are taken into account individually in the estimation of e.s.d.'s in distances, angles and torsion angles; correlations between e.s.d.'s in cell parameters are only used when they are defined by crystal symmetry. An approximate (isotropic) treatment of cell e.s.d.'s is used for estimating e.s.d.'s involving l.s. planes.
Refinement. Refinement of *F*^2^ against ALL reflections. The weighted *R*-factor *wR* and goodness of fit *S* are based on *F*^2^, conventional *R*-factors *R* are based on *F*, with *F* set to zero for negative *F*^2^. The threshold expression of *F*^2^ > σ(*F*^2^) is used only for calculating *R*-factors(gt) *etc*. and is not relevant to the choice of reflections for refinement. *R*-factors based on *F*^2^ are statistically about twice as large as those based on *F*, and *R*- factors based on ALL data will be even larger.

### Fractional atomic coordinates and isotropic or equivalent isotropic displacement parameters (Å^2^)

**Table:**

	*x*	*y*	*z*	*U*_iso_*/*U*_eq_	
S1	0.08799 (9)	0.63541 (4)	0.80960 (4)	0.0620 (2)	
O1	0.2717 (3)	0.63113 (17)	0.78264 (12)	0.0849 (6)	
O2	0.0096 (3)	0.55749 (13)	0.84498 (12)	0.0870 (7)	
N1	−0.0887 (3)	0.84795 (14)	0.70370 (13)	0.0566 (5)	
H1	−0.072 (4)	0.897 (2)	0.6945 (18)	0.073 (9)*	
C2	−0.2833 (3)	0.83013 (16)	0.71790 (15)	0.0520 (6)	
C3	−0.3323 (3)	0.73221 (16)	0.70025 (16)	0.0577 (6)	
H3A	−0.4623	0.7257	0.7074	0.069*	
H3B	−0.3078	0.7218	0.6464	0.069*	
C4	−0.2402 (3)	0.65759 (15)	0.74511 (17)	0.0589 (7)	
H4	−0.2719	0.6642	0.7993	0.071*	
N5	−0.0387 (3)	0.66325 (13)	0.73728 (11)	0.0524 (5)	
C6	0.0396 (3)	0.70241 (16)	0.66986 (13)	0.0497 (6)	
C7	0.1455 (3)	0.6493 (2)	0.62250 (16)	0.0659 (7)	
H7	0.1565	0.5877	0.6320	0.079*	
C8	0.2354 (4)	0.6879 (3)	0.56082 (18)	0.0855 (10)	
H8	0.3070	0.6524	0.5289	0.103*	
C9	0.2182 (4)	0.7779 (3)	0.54737 (17)	0.0862 (11)	
H9	0.2819	0.8041	0.5071	0.103*	
C10	0.1077 (4)	0.8310 (2)	0.59246 (15)	0.0676 (7)	
H10	0.0954	0.8922	0.5814	0.081*	
C11	0.0141 (3)	0.79417 (17)	0.65442 (13)	0.0501 (6)	
C12	−0.3967 (5)	0.8894 (2)	0.6654 (2)	0.0813 (9)	
H12A	−0.3735	0.9518	0.6767	0.122*	
H12B	−0.5231	0.8766	0.6732	0.122*	
H12C	−0.3652	0.8773	0.6130	0.122*	
C13	−0.3238 (4)	0.8554 (2)	0.80092 (17)	0.0704 (7)	
H13A	−0.2526	0.8182	0.8345	0.106*	
H13B	−0.4504	0.8460	0.8113	0.106*	
H13C	−0.2939	0.9177	0.8092	0.106*	
C14	−0.3082 (5)	0.5663 (2)	0.7171 (3)	0.0999 (13)	
H14A	−0.2821	0.5600	0.6635	0.150*	
H14B	−0.4369	0.5623	0.7251	0.150*	
H14C	−0.2485	0.5190	0.7451	0.150*	
C15	0.0750 (3)	0.72366 (16)	0.87704 (14)	0.0516 (5)	
C16	0.1482 (4)	0.80712 (19)	0.85876 (15)	0.0640 (7)	
H16	0.2035	0.8165	0.8115	0.077*	
C17	0.1378 (4)	0.8753 (2)	0.91126 (17)	0.0759 (8)	
H17	0.1873	0.9313	0.8988	0.091*	
C18	0.0569 (4)	0.8647 (2)	0.98198 (17)	0.0722 (7)	
C19	−0.0125 (4)	0.7808 (2)	0.99895 (18)	0.0796 (9)	
H19	−0.0666	0.7712	1.0464	0.095*	
C20	−0.0035 (4)	0.7106 (2)	0.94719 (17)	0.0700 (8)	
H20	−0.0508	0.6543	0.9600	0.084*	
C21	0.0455 (7)	0.9425 (3)	1.0377 (2)	0.1170 (14)	
H21A	−0.0147	0.9229	1.0836	0.176*	
H21B	−0.0217	0.9911	1.0150	0.176*	
H21C	0.1657	0.9628	1.0501	0.176*	

### Atomic displacement parameters (Å^2^)

**Table:**

	*U*^11^	*U*^22^	*U*^33^	*U*^12^	*U*^13^	*U*^23^
S1	0.0663 (4)	0.0509 (3)	0.0687 (4)	0.0183 (3)	0.0157 (3)	0.0095 (3)
O1	0.0646 (11)	0.1087 (16)	0.0812 (13)	0.0422 (12)	0.0166 (10)	0.0088 (13)
O2	0.1298 (19)	0.0453 (10)	0.0858 (13)	0.0116 (11)	0.0213 (13)	0.0147 (10)
N1	0.0579 (12)	0.0403 (11)	0.0715 (13)	−0.0081 (9)	0.0017 (11)	−0.0036 (10)
C2	0.0474 (12)	0.0439 (12)	0.0648 (15)	0.0013 (9)	−0.0032 (11)	−0.0035 (11)
C3	0.0432 (11)	0.0530 (13)	0.0770 (17)	−0.0057 (10)	0.0066 (12)	−0.0146 (13)
C4	0.0530 (13)	0.0434 (13)	0.0803 (17)	−0.0027 (11)	0.0239 (13)	−0.0049 (12)
N5	0.0497 (11)	0.0486 (11)	0.0591 (11)	0.0025 (8)	0.0130 (9)	−0.0015 (9)
C6	0.0443 (12)	0.0558 (13)	0.0491 (12)	−0.0020 (10)	0.0070 (10)	−0.0089 (10)
C7	0.0542 (14)	0.0773 (19)	0.0663 (15)	0.0042 (13)	0.0106 (12)	−0.0189 (15)
C8	0.0705 (19)	0.128 (3)	0.0583 (18)	−0.001 (2)	0.0171 (16)	−0.0234 (19)
C9	0.0677 (19)	0.140 (4)	0.0504 (16)	−0.031 (2)	0.0072 (14)	0.005 (2)
C10	0.0623 (16)	0.087 (2)	0.0541 (14)	−0.0195 (15)	−0.0074 (13)	0.0098 (14)
C11	0.0442 (11)	0.0583 (14)	0.0478 (12)	−0.0100 (10)	−0.0031 (10)	−0.0029 (11)
C12	0.081 (2)	0.0666 (17)	0.097 (2)	0.0052 (16)	−0.0252 (18)	0.0046 (16)
C13	0.0620 (15)	0.0673 (17)	0.0819 (18)	0.0051 (13)	0.0077 (14)	−0.0183 (16)
C14	0.077 (2)	0.0482 (15)	0.174 (4)	−0.0146 (14)	0.024 (2)	−0.018 (2)
C15	0.0412 (11)	0.0547 (13)	0.0590 (13)	0.0080 (10)	0.0036 (11)	0.0126 (11)
C16	0.0731 (17)	0.0651 (17)	0.0538 (14)	−0.0065 (14)	−0.0021 (13)	0.0170 (13)
C17	0.091 (2)	0.0607 (17)	0.0764 (19)	−0.0149 (15)	−0.0173 (16)	0.0148 (15)
C18	0.0755 (18)	0.0681 (18)	0.0730 (17)	0.0079 (16)	−0.0034 (15)	−0.0024 (16)
C19	0.079 (2)	0.092 (2)	0.0674 (17)	0.0025 (17)	0.0194 (16)	−0.0025 (17)
C20	0.0712 (17)	0.0671 (18)	0.0717 (17)	−0.0057 (14)	0.0213 (15)	0.0072 (14)
C21	0.144 (4)	0.103 (3)	0.104 (3)	0.019 (3)	−0.017 (3)	−0.032 (2)

### Geometric parameters (Å, °)

**Table:**

S1—O2	1.430 (2)	C10—C11	1.394 (3)
S1—O1	1.434 (2)	C10—H10	0.9300
S1—N5	1.623 (2)	C12—H12A	0.9600
S1—C15	1.761 (3)	C12—H12B	0.9600
N1—C11	1.395 (3)	C12—H12C	0.9600
N1—C2	1.478 (3)	C13—H13A	0.9600
N1—H1	0.75 (3)	C13—H13B	0.9600
C2—C12	1.519 (4)	C13—H13C	0.9600
C2—C13	1.526 (4)	C14—H14A	0.9600
C2—C3	1.525 (3)	C14—H14B	0.9600
C3—C4	1.515 (4)	C14—H14C	0.9600
C3—H3A	0.9700	C15—C20	1.368 (4)
C3—H3B	0.9700	C15—C16	1.385 (4)
C4—N5	1.493 (3)	C16—C17	1.366 (4)
C4—C14	1.522 (4)	C16—H16	0.9300
C4—H4	0.9800	C17—C18	1.380 (4)
N5—C6	1.433 (3)	C17—H17	0.9300
C6—C7	1.382 (3)	C18—C19	1.375 (4)
C6—C11	1.397 (3)	C18—C21	1.510 (5)
C7—C8	1.387 (5)	C19—C20	1.379 (4)
C7—H7	0.9300	C19—H19	0.9300
C8—C9	1.359 (5)	C20—H20	0.9300
C8—H8	0.9300	C21—H21A	0.9600
C9—C10	1.378 (5)	C21—H21B	0.9600
C9—H9	0.9300	C21—H21C	0.9600
			
O2—S1—O1	119.16 (14)	N1—C11—C10	121.6 (2)
O2—S1—N5	107.96 (13)	N1—C11—C6	120.6 (2)
O1—S1—N5	107.36 (11)	C10—C11—C6	117.6 (2)
O2—S1—C15	106.71 (12)	C2—C12—H12A	109.5
O1—S1—C15	107.67 (14)	C2—C12—H12B	109.5
N5—S1—C15	107.48 (10)	H12A—C12—H12B	109.5
C11—N1—C2	121.86 (19)	C2—C12—H12C	109.5
C11—N1—H1	109 (2)	H12A—C12—H12C	109.5
C2—N1—H1	112 (3)	H12B—C12—H12C	109.5
N1—C2—C12	109.2 (2)	C2—C13—H13A	109.5
N1—C2—C13	107.8 (2)	C2—C13—H13B	109.5
C12—C2—C13	108.9 (2)	H13A—C13—H13B	109.5
N1—C2—C3	111.42 (19)	C2—C13—H13C	109.5
C12—C2—C3	107.3 (2)	H13A—C13—H13C	109.5
C13—C2—C3	112.2 (2)	H13B—C13—H13C	109.5
C4—C3—C2	118.8 (2)	C4—C14—H14A	109.5
C4—C3—H3A	107.6	C4—C14—H14B	109.5
C2—C3—H3A	107.6	H14A—C14—H14B	109.5
C4—C3—H3B	107.6	C4—C14—H14C	109.5
C2—C3—H3B	107.6	H14A—C14—H14C	109.5
H3A—C3—H3B	107.0	H14B—C14—H14C	109.5
N5—C4—C3	110.93 (19)	C20—C15—C16	119.8 (3)
N5—C4—C14	110.3 (2)	C20—C15—S1	121.1 (2)
C3—C4—C14	109.5 (3)	C16—C15—S1	119.09 (19)
N5—C4—H4	108.7	C17—C16—C15	118.9 (3)
C3—C4—H4	108.7	C17—C16—H16	120.6
C14—C4—H4	108.7	C15—C16—H16	120.6
C6—N5—C4	119.9 (2)	C16—C17—C18	122.7 (3)
C6—N5—S1	120.68 (16)	C16—C17—H17	118.7
C4—N5—S1	119.08 (17)	C18—C17—H17	118.7
C7—C6—C11	120.9 (2)	C19—C18—C17	117.2 (3)
C7—C6—N5	119.2 (2)	C19—C18—C21	121.9 (3)
C11—C6—N5	119.8 (2)	C17—C18—C21	120.9 (3)
C6—C7—C8	120.0 (3)	C18—C19—C20	121.5 (3)
C6—C7—H7	120.0	C18—C19—H19	119.3
C8—C7—H7	120.0	C20—C19—H19	119.3
C9—C8—C7	119.6 (3)	C15—C20—C19	120.0 (3)
C9—C8—H8	120.2	C15—C20—H20	120.0
C7—C8—H8	120.2	C19—C20—H20	120.0
C8—C9—C10	121.0 (3)	C18—C21—H21A	109.5
C8—C9—H9	119.5	C18—C21—H21B	109.5
C10—C9—H9	119.5	H21A—C21—H21B	109.5
C9—C10—C11	120.8 (3)	C18—C21—H21C	109.5
C9—C10—H10	119.6	H21A—C21—H21C	109.5
C11—C10—H10	119.6	H21B—C21—H21C	109.5
			
C11—N1—C2—C12	−95.9 (3)	C8—C9—C10—C11	1.7 (4)
C11—N1—C2—C13	145.9 (2)	C2—N1—C11—C10	123.8 (3)
C11—N1—C2—C3	22.4 (3)	C2—N1—C11—C6	−62.0 (3)
N1—C2—C3—C4	61.1 (3)	C9—C10—C11—N1	175.7 (2)
C12—C2—C3—C4	−179.4 (2)	C9—C10—C11—C6	1.3 (4)
C13—C2—C3—C4	−59.8 (3)	C7—C6—C11—N1	−178.1 (2)
C2—C3—C4—N5	−57.2 (3)	N5—C6—C11—N1	−0.7 (3)
C2—C3—C4—C14	−179.3 (2)	C7—C6—C11—C10	−3.7 (3)
C3—C4—N5—C6	−27.7 (3)	N5—C6—C11—C10	173.7 (2)
C14—C4—N5—C6	93.9 (3)	O2—S1—C15—C20	−1.4 (3)
C3—C4—N5—S1	145.32 (18)	O1—S1—C15—C20	−130.5 (2)
C14—C4—N5—S1	−93.1 (3)	N5—S1—C15—C20	114.2 (2)
O2—S1—N5—C6	−146.99 (18)	O2—S1—C15—C16	177.6 (2)
O1—S1—N5—C6	−17.3 (2)	O1—S1—C15—C16	48.5 (2)
C15—S1—N5—C6	98.24 (18)	N5—S1—C15—C16	−66.8 (2)
O2—S1—N5—C4	40.06 (19)	C20—C15—C16—C17	−1.0 (4)
O1—S1—N5—C4	169.70 (18)	S1—C15—C16—C17	−180.0 (2)
C15—S1—N5—C4	−74.71 (19)	C15—C16—C17—C18	−0.1 (4)
C4—N5—C6—C7	−116.8 (2)	C16—C17—C18—C19	1.0 (4)
S1—N5—C6—C7	70.3 (3)	C16—C17—C18—C21	−178.9 (3)
C4—N5—C6—C11	65.7 (3)	C17—C18—C19—C20	−0.8 (5)
S1—N5—C6—C11	−107.2 (2)	C21—C18—C19—C20	179.1 (3)
C11—C6—C7—C8	3.2 (4)	C16—C15—C20—C19	1.2 (4)
N5—C6—C7—C8	−174.3 (2)	S1—C15—C20—C19	−179.8 (2)
C6—C7—C8—C9	−0.1 (4)	C18—C19—C20—C15	−0.3 (5)
C7—C8—C9—C10	−2.3 (5)		

### Hydrogen-bond geometry (Å, °)

**Table:**

*D*—H···*A*	*D*—H	H···*A*	*D*···*A*	*D*—H···*A*
C20—H20···O2	0.93	2.51	2.885 (4)	105
N1—H1···O2^i^	0.75 (3)	2.52 (3)	3.268 (3)	176 (3)
C14—H14B···O1^ii^	0.96	2.58	3.436 (4)	149
C13—H13A···Cg2	0.96	2.90	3.7592 (31)	150
C19—H19···Cg2^i^	0.93	2.90	3.5192 (34)	126

Symmetry codes:  (i) −*x*, *y*+1/2, −*z*+3/2; (ii) *x*−1, *y*, *z*.
